# Race, Ethnicity, Insurance Payer, and Pediatric Cardiac Arrest Survival

**DOI:** 10.1001/jamanetworkopen.2025.31213

**Published:** 2025-09-10

**Authors:** Amanda J. O’Halloran, Garrett Keim, Cody-Aaron Gathers, Jessica Fowler, Maryam Y. Naim, Joseph W. Rossano, Robert A. Berg, Robert M. Sutton, Nadir Yehya, Ryan W. Morgan

**Affiliations:** 1 Division of Critical Care Medicine, Department of Anesthesiology and Critical Care, The Children’s Hospital of Philadelphia, Philadelphia, Pennsylvania; 2 Department of Anesthesiology and Critical Care, University of Pennsylvania School of Medicine, Philadelphia; 3 Leonard Davis Institute of Economics, University of Pennsylvania, Philadelphia; 4 Division of Cardiology, Department of Pediatrics, The Children's Hospital of Philadelphia, Philadelphia, Pennsylvania; 5 Division of Cardiac Critical Care Medicine, Department of Anesthesiology and Critical Care, The Children's Hospital of Philadelphia, Philadelphia, Pennsylvania; 6 Department of Pediatrics, University of Pennsylvania School of Medicine, Philadelphia

## Abstract

**Importance:**

Lower survival rates among Black adults relative to White adults after in-hospital cardiac arrest are well-described, but these findings have not been consistently replicated in pediatric studies.

**Objective:**

To use a large, national, population-based inpatient database to evaluate the associations between in-hospital mortality in children receiving cardiopulmonary resuscitation (CPR) and patient race or ethnicity, patient insurance status, and the treating hospital’s proportion of Black and publicly insured patients.

**Design, Setting, and Participants:**

This retrospective population-based cohort study used the Healthcare Cost and Utilization Project Kids’ Inpatient Database (1997-2019 triennial versions). Participants included pediatric inpatients (aged <18 years) who received in-hospital CPR. Initial data analysis occurred January 20 to July 31, 2023. Revision analyses were completed December 1, 2024, to March 3, 2025.

**Exposures:**

The exposures for the primary analyses were race or ethnicity and insurance payer. The secondary analyses exposures were the proportion of the treating hospital’s admissions (not limited to those receiving CPR) of Black patients and of publicly insured patients.

**Main Outcomes and Measures:**

The primary outcome for all analyses was in-hospital mortality.

**Results:**

The final cohort included 27 332 children (6366 neonates aged 5-28 days [23.3%], 9665 infants aged 29 days to <1 year [35.4%], 4867 aged 1 year to <8 years [17.8%], and 6434 aged ≥8 years [23.5%]; 15 356 male [56.2%]; 6081 (22.2%) Black; 5123 (18.7%) Hispanic; 13 062 (47.8%) White; and 3066 (11.2%) other race or ethnicity) who received in-hospital CPR at sites with 3899 unique hospital identification numbers. Relative to White children, higher odds of in-hospital mortality were observed for Black (adjusted odds ratio [AOR], 1.20; 95% CI, 1.08-1.34; *P* < .001) and Hispanic (AOR, 1.16; 95% CI, 1.04-1.30; *P* = .006) children and those of other race or ethnicity (AOR, 1.37; 95% CI 1.20-1.58; *P* < .001). Public insurance was not associated with in-hospital mortality compared with private insurance (AOR, 1.00; 95% CI, 0.91-1.11; *P* = .93). On multivariable analysis of the 2003-2019 datasets, children receiving CPR at hospitals with the highest proportion of Black patients (>30.1%) had higher odds of in-hospital mortality than children receiving CPR at hospitals with the lowest proportion of Black patients (AOR, 1.50; 95% CI, 1.17-1.92; *P* = .001).

**Conclusions and Relevance:**

In this retrospective cohort study of pediatric in-hospital cardiac arrest in a large, national, administrative dataset, children of racial and ethnic minority groups receiving CPR had higher odds of in-hospital mortality. In addition, the odds of in-hospital mortality among children receiving CPR were higher at hospitals with the highest proportion of Black patients.

## Key Points

**Question:**

Are race and ethnicity or insurance payer associated with survival after pediatric in-hospital cardiopulmonary resuscitation (CPR) in the US?

**Findings:**

In this cohort study of 27 332 children receiving in-hospital CPR, children from racial and ethnic minority groups had significantly higher odds of in-hospital mortality. Children receiving CPR at hospitals treating the highest proportion of Black patients had 50% higher odds of in-hospital mortality than children receiving CPR at hospitals with the lowest proportion of Black patients.

**Meaning:**

These findings suggest that elucidation of the mechanisms underlying differences in outcomes of pediatric in-hospital cardiac arrest is needed.

## Teaser

This cohort study evaluates the associations between mortality among children receiving in-hospital cardiopulmonary resuscitation and patient race or ethnicity, patient insurance payer, and the treating hospital’s proportion of Black patients.

## Introduction

Cardiac arrest disparities have been well-described among racial, ethnic, and socioeconomic groups. Disparate bystander cardiopulmonary resuscitation (CPR) rates have been reported for out-of-hospital cardiac arrest,^1,2,3,4^ and survival rates of in-hospital cardiac arrest (IHCA) are lower in Black adults than White adults. Differences in adult outcomes are partially attributable to hospital characteristics (eg, nursing staffing) and resuscitation quality (eg, time to defibrillation).^4,5,6,7,8^ The sole systematic pediatric IHCA investigation of racial disparities using the American Heart Association’s Get With the Guidelines–Resuscitation (GWTG-R) registry identified an association between Black race and lower rates of return of spontaneous circulation compared with White race (70% vs 75%) but did not find an association between race and survival to discharge.^9^

While the lack of an association between race and survival in previous pediatric work is encouraging, the generalizability may be limited by the small patient population (eg, <900 Black patients) and characteristics of sites enrolled in the GWTG-R, a voluntary quality improvement registry. Moreover, exploration of the association between socioeconomic status and pediatric IHCA outcomes remains limited to date. A better understanding of this knowledge gap could provide insight into potentially modifiable differences in pediatric IHCA care.

We therefore used a large, nationally representative inpatient database to evaluate associations between mortality among children receiving in-hospital CPR and (1) patient race or ethnicity, (2) patient insurance payer, (3) the treating hospital’s proportion of Black patients, and (4) the treating hospital’s proportion of publicly insured patients. We hypothesized that among children receiving CPR, Black race and public insurance would be associated with increased mortality. As worse adult IHCA outcomes have been described in hospitals with large populations of Black patients,^10^ we focused on proportion of Black patients as a hospital-level metric that may provide insight into a mechanism underlying pediatric IHCA differences.

## Methods

### Data Source

This retrospective cohort study used the 1997, 2000, 2003, 2006, 2009, 2012, 2016, and 2019 releases of the Healthcare Cost and Utilization Project Kids’ Inpatient Database (KID), a large, national (US), inpatient administrative database.^11^ KID includes demographic characteristics, diagnoses, procedures, insurance status, and hospital characteristics from a sample of pediatric (aged <21 years) discharges. National estimates are generated using sampling weights that adjust for the survey design, ensuring representation of pediatric hospitalizations across hospital types and geographic regions.^11^ Each weighted KID release represents approximately 7 million pediatric hospitalizations.

The Children’s Hospital of Philadelphia Institutional Review Board determined that this protocol was not human participants research and did not require informed consent. We followed the Strengthening the Reporting of Observational Studies in Epidemiology (STROBE) guidelines.

### Study Population

Patients younger than 18 years with a nonobstetric illness category and who were not newborn infants were eligible (eTable 1 in Supplement 1). Since newborn patients requiring resuscitation have unique IHCA characteristics^12^ and different clinical teams than other events, we excluded events with newborn admission type and length of stay less than 5 days. Children receiving in-hospital CPR were identified using procedure codes from the *International Classification of Diseases, Ninth Revision*, and *International and Statistical Classification of Diseases and Related Health Problems, Tenth Revision* (eTable 1 in Supplement 1). Using procedure codes (rather than diagnosis codes) ensured that CPR occurred during the hospitalization and was not related to a preceding out-of-hospital cardiac arrest or one that occurred during a previous admission. Participants missing a primary exposure (race and ethnicity or insurance payer) or the outcome (in-hospital mortality) were excluded. Post hoc analyses of excluded patients are in eAppendix 1 in Supplement 1. Children with a disposition of transfer to another acute care hospital were excluded from the primary analysis since they could not experience the outcome for that admission (ie, their discharge as alive or deceased after receiving in-hospital CPR would be reported by the subsequent facility).

### Study Variables

The exposures for the primary analyses were race or ethnicity and insurance payer (modeled together). Race and ethnicity are reported as a combined variable in KID, with the following categories: American Indian or Alaska Native, Asian or Pacific Islander, Black, Hispanic, White, and other. Since each hospital provides separately collected race and ethnicity data, information on how individuals are classified is unavailable. The racial and ethnic categories denoted as other in KID are not delineated. For our analyses, American Indian or Alaska Native, Asian or Pacific Islander, and other were combined into a category of other. Insurance payer categories in KID include Medicare, Medicaid, private (including health maintenance organizations), self-pay, no charge, and other. For our analyses, Medicare and Medicaid were combined into a single category (public insurance). Self-pay, no charge, and other were combined into a category of other. The primary outcome for all analyses was in-hospital mortality.

### Statistical Analysis

Initial data analysis occurred from January 20 to July 31, 2023. Revision analyses were completed from December 1, 2024, to March 3, 2025. Patient characteristics were compared based on survival status using Wilcoxon rank-sum testing for continuous variables and Pearson χ^2^ testing for categorical variables. All analyses were evaluated at a 2-sided significance level of *P* ≤ .05 using Stata, version 18 (StataCorp).

#### Model Development

We developed a weighted multivariable mixed-effect logistic regression model to assess associations between in-hospital mortality and the exposures of race and ethnicity and insurance payer. Patient and hospital characteristics were considered fixed effects and admitting hospital was used as a random effect. Covariates were selected a priori based on known or hypothesized associations with the exposure(s) and outcome (eFigure 1 in Supplement 1). They included age at admission (neonates [5-28 days], infants [29 days to <1 year], younger children [1 to <8 years], and older children [≥8 years])^13^; sex; preexisting kidney insufficiency and cancer^14^; and neighborhood income (categorized into quartiles based on median household income by zip code). Hospital characteristics of interest were region (Northeast, Midwest, South, or West), urban location, teaching status, and size (small, medium, or large).

To determine whether hospital racial and ethnic composition was part of the association between race and ethnicity and pediatric IHCA mortality, an alternative model was developed that incorporated the treating hospital’s percentage of admissions occurring in Black patients (derivation described later) as an additional fixed effect. We performed stratified secondary analyses (1) by region, (2) by KID version year, and (3) by including patients with a disposition of transfer to another acute care facility into the cohort as survivors—all using the same weighted mixed-effect regression procedures.

#### Joint Exposure Analysis

Joint exposure modeling has been used previously to analyze combined associations of 2 or more exposures with an outcome of interest.^15,16^ We created a joint exposure variable combining race and ethnicity, median neighborhood income (above vs below median), and insurance payer with the reference group of White race, public insurance, and below median income. Neighborhood income was included as an additional proxy for social determinants of health, since more comprehensive measures (eg, Childhood Opportunity Index) are unavailable in KID.^17^ The joint exposure variable was used in a weighted mixed-effect logistic regression model with the previously stated fixed and random effects.

#### Association Between Patient Composition of the Treating Hospital and Mortality After IHCA

To determine whether hospital patient racial and ethnic composition or hospital payer mix (as a proxy for institutional resources) was associated with IHCA mortality, categorical variables of the percentage of admissions occurring among Black patients (for the race and ethnicity composition analysis) and publicly insured patients (for the insurance payer mix analysis) were derived. Using the full KID (not limited to those receiving CPR; triennial releases from 2003-2019), the percentage of Black patients and of publicly insured patients was determined for each unique center identification number (ID). Of note, hospital IDs are reassigned for each KID publication, preventing tracking hospitals across release years.

Hospitals were divided into quintiles based on percentage of overall admissions occurring among Black patients (0%-3.00% [reference group], 3.01%-7.00%, 7.01%-15.00%, 15.01%-30.00%, and >30.00%) and by percentage occurring among publicly insured patients (0%-40.90% [reference group], 40.91%-53.50%, 53.51%-64.10%, and >64.10%). For both race and ethnicity and payer mix, odds of in-hospital mortality after CPR were compared between these quintiles using the same weighted mixed-effect regression procedures as those from the primary analysis.

## Results

### Description of Cohort

**Figure 1.**
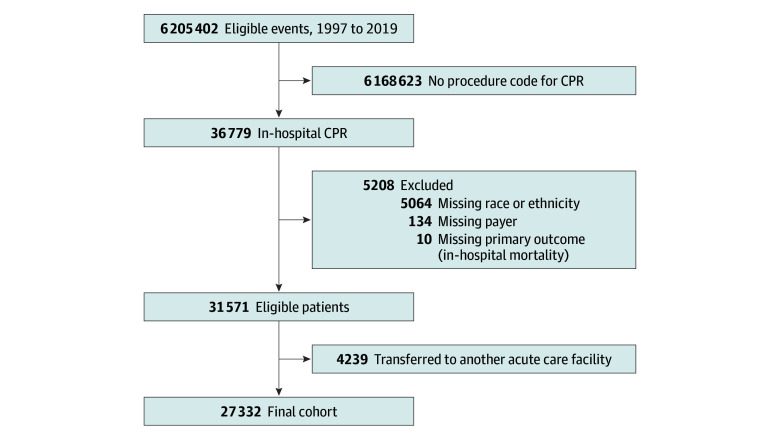
Study Flow Diagram CPR indicates cardiopulmonary resuscitation.

We identified 6 205 402 unweighted eligible admissions. After excluding events that did not include in-hospital CPR (n = 6 168 623), those missing a primary exposure or the outcome (n = 5208) (eAppendix 1 in Supplement 1), or those transferred to another acute care facility (n = 4239), the cohort included 27 332 patients at 3899 centers with unique hospital IDs (Figure 1). A total of 6366 patients (23.3%) were neonates; 9665 (35.4%), infants; 4867 (17.8%), 1 to younger than 8 years; and 6434 (23.5%), 8 years or older. A total of 11 976 patients (43.8%) were female and 15 356 (56.2%) were male (Table 1). For race or ethnicity, 6081 patients (22.2%) were Black, 5123 (18.7%) were Hispanic, 13 062 (47.8%) were White, and 3066 (11.2%) were other race or ethnicity. Most patients had public insurance (13 689 [50.1%]), followed by private (11 010 [40.3%]), and other (2633 [9.6%]). Most patients (5277 of 6919 [76.3%]) had at least 1 complex chronic condition,^14^ and the majority were admitted to large (14 967 of 24 203 [61.8%]), urban (23 285 of 24 203 [96.2%]), and/or teaching institutions (19 197 of 24 203 [79.3%]). The rate of in-hospital mortality was 40.5%. Cohort characteristics by race and ethnicity and insurance payer are shown in eTables 2 and 3 in Supplement 1.

**Table 1.** Patient and Hospital Characteristics, Stratified by Mortality

Characteristic	Patient group, No. (%)			*P* value
	Total (N = 27 332)	Survived to hospital discharge (n = 16 252)	In-hospital mortality (n = 11 080)	
Age				
5-28 d	6366 (23.3)	3504 (21.6)	2862 (25.8)	<.001
29 d to <1 y	9665 (35.4)	5267 (32.4)	4398 (39.7)	
1 y to <8 y	4867 (17.8)	3306 (20.3)	1561 (14.1)	
≥8 y	6434 (23.5)	4175 (25.7)	2259 (20.4)	
Sex				
Female	11 976 (43.8)	7144 (44.0)	4832 (43.6)	.63
Male	15 356 (56.2)	9108 (56.0)	6248 (56.4)	
Race and ethnicity				
Black	6081 (22.2)	3387 (20.8)	2694 (24.3)	<.001
Hispanic	5123 (18.7)	2702 (16.6)	2421 (21.9)	
White	13 062 (47.8)	8531 (52.5)	4531 (40.9)	
Other^a^	3066 (11.2)	1632 (10.0)	1434 (12.9)	
Insurance payer				
Public	13 689 (50.1)	8020 (49.3)	5669 (51.2)	<.001
Private	11 010 (40.3)	6906 (42.5)	4104 (37.0)	
Other^b^	2633 (9.6)	1326 (8.2)	1307 (11.8)	
Complex chronic condition^c^	5277 (76.3)	2396 (66.5)	2881 (86.9)	<.001
Admission day is a weekend^d^	5528 (21.5)	2861 (18.5)	2667 (25.9)	<.001
Length of stay, median (IQR), d	4 (1-15)	6 (3-22)	1 (0-8)	<.001
Patient disposition				
Discharged to home or self-care	13 947 (51.0)	13 947 (85.8)	0	<.001
Other transfer (SNF or intermediate care)	732 (2.7)	732 (4.5)	0	
Discharged to home health care	1534 (5.6)	1534 (9.4)	0	
Left against medical advice	16 (0.1)	16 (0.1)	0	
In-hospital mortality	11 080 (40.5)	0	11 080 (100)	
Discharged alive, destination unknown	23 (0.1)	23 (0.1)	0	
Total charges, median (IQR), US $	36 747 (11 200-156 332)	35 804 (11 041-180 688)	37 817 (11 598-129 155)	<.001
Median household income quartile for patient zip code^e^				
0-25th Percentile	8771 (33.0)	5270 (33.4)	3501 (32.5)	.14
26th-50th Percentile	6692 (25.2)	3914 (24.8)	2778 (25.8)	
51st-75th Percentile	5822 (21.9)	3441 (21.8)	2381 (22.1)	
76th-100th Percentile	5274 (19.9)	3173 (20.1)	2101 (19.5)	
Hospital region				
Northeast	3931 (14.4)	2188 (13.5)	1743 (15.7)	<.001
Midwest	4314 (15.8)	2453 (15.1)	1861 (16.8)	
South	12 310 (45.0)	8038 (49.5)	4272 (38.6)	
West	6777 (24.8)	3573 (22.0)	3204 (28.9)	
Hospital urban location^f^	23 285 (96.2)	12 717 (95.5)	10 568 (97.1)	<.001
Teaching hospital^f^	19 197 (79.3)	10 473 (78.6)	8724 (80.1)	.004
Hospital size^f^^,^^g^				
Small	3651 (15.1)	2453 (18.4)	1198 (11.0)	<.001
Medium	5585 (23.1)	3101 (23.3)	2484 (22.8)	
Large	14 967 (61.8)	7763 (58.3)	7204 (66.2)	

Abbreviation: SNF, skilled nursing facility.

^a^ Includes American Indian or Alaska Native, Asian or Pacific Islander, and other categorizations in the Healthcare Cost and Utilization Project Kids’ Inpatient Database.

^b^ Includes self-pay, no charge, and other.

^c^ Owing to missing data, includes 6919 patients.

^d^ Owing to missing data, includes 25 721 patients.

^e^ Owing to missing data, includes 26 559 patients.

^f^ Owing to missing data, includes 24 203 patients.

^g^ Hospital size in the database takes into account the hospital’s region, urban-rural designation, and teaching status.

### Association of Race and Ethnicity With In-Hospital Mortality

**Figure 2.**
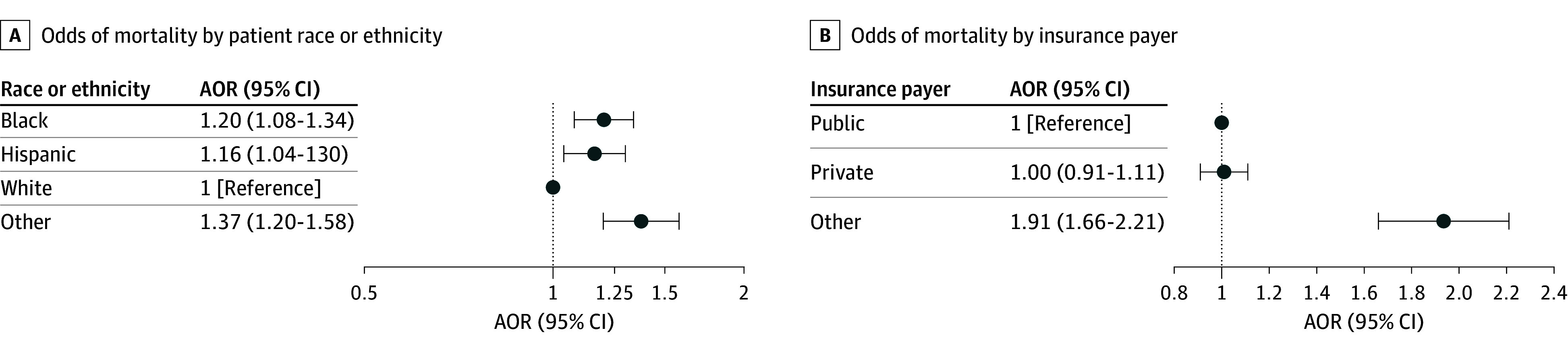
Forest Plot of Adjusted Odds of In-Hospital Mortality After Cardiopulmonary Resuscitation (CPR) Adjusted odds ratios (AORs) are identified by patient race or ethnicity and by insurance payer. Other race includes American Indian or Alaska Native, Asian or Pacific Islander, and other categorizations in the Healthcare Cost and Utilization Project Kids’ Inpatient Database (KID). Public insurance payer status includes Medicare and Medicaid. Other insurance payer status includes self-pay, no charge, and other. Hospital (size, region, teaching, rural or urban distinction, and KID cohort year) and patient-level factors (age, presence of chronic kidney insufficiency or cancer, income quartile by zip code, insurance payer, and biological sex) were considered fixed effects and treating hospital was considered a random effect.

Compared with White children, the unadjusted odds of in-hospital mortality was higher for children of Black (odds ratio [OR], 1.28; 95% CI, 1.17-1.49; *P* < .001), Hispanic (OR, 1.23; 95% CI, 1.13-1.35; *P* < .001), and other (OR, 1.32; 95% CI, 1.19-1.47; *P* < .001) race or ethnicity. In the multivariable analysis, these associations remained (adjusted OR [AOR] for Black, 1.20 [95% CI, 1.08-1.34; *P* < .001]; AOR for Hispanic, 1.16 [95% CI, 1.04-1.30; *P* = .008]; and AOR for other, 1.37 [95% CI, 1.20-1.58; *P* < .001]) (Figure 2). Model diagnostics are found in eAppendix 2 in Supplement 1.

The secondary analysis that included patients transferred to other acute care facilities in the cohort as survivors also demonstrated higher in-hospital mortality for racial and ethnic minority categories (AOR for Black, 1.20 [95% CI, 1.08-1.33; *P* = .001]; AOR for Hispanic, 1.18 [95% CI, 1.06-1.31; *P* = .003]; and AOR for other, 1.33 [95% CI, 1.17-1.50; *P* < .001]) (eTable 4 in Supplement 1). The alternative model that incorporated the treating hospital’s percentage of Black admissions resulted in partial attenuation of the mortality odds for Black patients, with minimal difference on AORs for Hispanic and other patients (eTable 5 in Supplement 1).

### Association of Insurance Payer With In-Hospital Mortality

On unadjusted analysis, compared with children with public insurance, the odds of in-hospital mortality were higher among those with other insurance payer (OR, 1.74; 95% CI, 1.55-1.95; *P* < .001) and lower among those with private insurance (OR, 0.93; 95% CI, 0.87-1.00; *P* = .04). In the multivariable analysis, the odds of in-hospital mortality in children receiving CPR was higher for children with other insurance payer compared to those with public insurance (AOR, 1.91; 95% CI, 1.66-2.21; *P* < .001) but was not associated with private compared with public insurance (AOR, 1.00; 95% CI, 0.91-1.11; *P* = .93) (Figure 2 and eAppendix 2 in Supplement 1).

### Secondary Analyses Stratified by Region and KID Release Year

On multivariable analysis stratified by region, children with other race and ethnicity had higher odds of in-hospital mortality in all regions compared with White children (AOR for Northeast, 1.38 [95% CI, 1.20-1.58]; AOR for Midwest, 1.53 [95% CI, 1.12-2.09]; AOR for South, 1.33 [95% CI, 1.05-1.68]; AOR for West, 1.38 [95% CI, 1.08-1.77]). Mortality differences relative to White children remained for all groups in the Northeast (AOR for Black, 1.20 [95% CI, 1.08-1.34]; AOR for Hispanic, 1.16 [95% CI, 1.04-1.30]; AOR for other, 1.38 [95% CI, 1.20-1.58]) and for Hispanic children in the West (AOR, 1.26; 95% CI, 1.06-1.50) (eFigure 2 in Supplement 1).

In the most recent releases of the KID (2016 and 2019), all racial and ethnic minority groupings were associated with higher odds of in-hospital mortality (eFigure 3 in Supplement 1). Black children had 46% and 45% higher odds of in-hospital mortality compared with White children (AORs, 1.46 [95% CI, 1.21-1.78] and 1.45 [95% CI, 1.22-1.74], respectively).

### Joint Model

**Figure 3.**
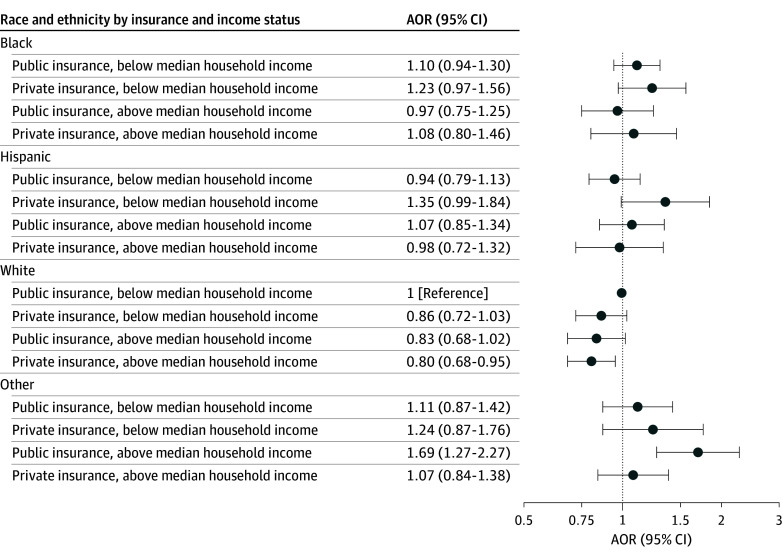
Forest Plot of Joint Association of Race or Ethnicity, Insurance Payer, and Median Household Income With In-Hospital Mortality Other race includes American Indian or Alaska Native, Asian or Pacific Islander, and other categorizations in the Healthcare Cost and Utilization Project Kids’ Inpatient Database (KID). Public insurance payer status includes Medicare and Medicaid. Hospital (size, region, teaching, rural or urban distinction, and KID cohort year) and patient-level factors (age, presence of chronic kidney insufficiency or cancer, income quartile by zip code, insurance payer, and biological sex) were considered fixed effects and treating hospital was considered a random effect. AOR indicates adjusted odds ratio.

In comparison with the reference group (White race, public insurance, below median neighborhood income), the odds of in-hospital mortality were lowest among White patients with private insurance who lived in a zip code with above-median neighborhood income (AOR, 0.80; 95% CI, 0.68-0.95) (Figure 3). In comparison with the reference group, the odds of in-hospital mortality were higher among patients with other race or ethnicity, public insurance, and above-median household income (AOR, 1.69; 95% CI, 1.27-2.27). The odds of in-hospital mortality did not differ significantly for any other groupings.

### Odds of Mortality by Treating Hospital’s Proportions of Black and Publicly Insured Patients

Across the 3899 unique hospital IDs that contributed patients to the cohort, the percentage of overall hospital admissions occurring among Black patients ranged from 1% to 98.4% (median, 17.9%). Patients receiving CPR at hospitals caring for the highest proportion of Black patients had a higher adjusted odds of in-hospital mortality (AOR, 1.50; 95% CI, 1.17-1.92; *P* = .001) compared with patients at hospitals caring for the lowest proportion of Black patients (Table 2). Patients receiving CPR at hospitals caring for the highest proportion of publicly insured patients (>64.11%) had a higher adjusted odds of in-hospital mortality (AOR, 1.21; 95% CI, 1.02-1.42; *P* = .03) compared with patients at hospitals caring for the lowest proportion of publicly insured patients (eTable 6 in Supplement 1).

**Table 2.** Adjusted Odds of In-Hospital Mortality After CPR by Treating Hospital’s Proportion of Black Patients^a^

Proportion of Black patients at treating hospital, %	Hospitals, No.	AOR (95% CI)	*P* value
0-3.00	971	1 [Reference]	NA
3.01-7.00	638	1.16 (0.93-1.45)	.19
7.01-15.00	899	1.18 (0.96-1.45)	.11
15.01-30.00	910	1.16 (0.94-1.45)	.17
>30.00	694	1.50 (1.17-1.92)	.001

Abbreviations: AOR, adjusted odds ratio; CPR, cardiopulmonary resuscitation; NA, not applicable.

^a^ Includes data from 2003 to 2019. Hospital (size, region, teaching, rural or urban distinction, and Healthcare Cost and Utilization Project Kids’ Inpatient Database cohort year) and patient-level factors (age, presence of chronic kidney insufficiency or cancer, income quartile by zip code, insurance payer, and biological sex) level factors were considered fixed effects and treating hospital was considered a random effect.

## Discussion

In this cohort study, we investigated associations of race and ethnicity and insurance payer with survival to discharge after pediatric in-hospital CPR using the KID, a large, national, administrative database. After adjusting for key confounders, children in racial or ethnic minority groups had lower odds of survival. While the odds of survival did not differ between children with public and private insurance, those with other insurance (self-pay, no insurance, or other) were less likely to survive to discharge. Children receiving CPR at hospitals treating higher proportions of Black patients (>30%) had 50% higher odds of in-hospital mortality than children receiving CPR at hospitals caring for the lowest proportion of Black patients (0%-3%). Collectively, these data provide a novel appraisal of important differences in pediatric IHCA outcomes.

Health care disparities are widespread and important.^18^ Specifically among critically ill children, articles since 2021 have highlighted the prevalence of disparities driven by socioeconomic and racial or ethnic inequities.^19,20^ Notable examples include lower bystander CPR rates for Black and Hispanic children and higher illness severity at pediatric intensive care unit admission among uninsured children. In contrast to the limited previously published data,^9^ our study identified lower odds of survival after in-hospital CPR for children from minoritized racial or ethnic categorizations. In the context of the body of evidence describing pervasive health care disparities among vulnerable children, this finding highlights the need to better characterize these differences and their underlying determinants.

Understanding mechanisms contributing to these differences is necessary for mitigation. We used joint modeling, which can help disentangle the complex influence of socioeconomic factors on outcomes, to better understand the interplay of race or ethnicity, neighborhood income, and insurance payer.^15,16^ For example, if each race and ethnicity had a similar pattern of increased mortality for patients from zip codes with lower median income, that could suggest that neighborhood income may be the driver. The results of our model suggest that complex interactions between patient and hospital factors influence IHCA survival differences. Living in a higher-income neighborhood was protective for White, Black, and Hispanic children, but those in the other race or ethnicity category had increased odds of mortality if they had public insurance and lived in one of these areas. Future investigation exploring more granular household resource data and neighborhood characteristics may improve our understanding of these findings.

The increased risk of post-CPR mortality among patients from racial and ethnic minority groups may be associated with a host of societal, hospital, and/or individual factors. In addition to evaluating patient-level differences, we explored associations between overall hospital percentage of Black patients and publicly insured patients and the mortality rates in children receiving in-hospital CPR and found that the odds of mortality were significantly higher among patients at hospitals caring for the highest proportions of Black and publicly insured patients. A similar association has been shown between hospital proportion of Black patients and adult IHCA mortality.^10^ These findings suggest that hospital differences in resuscitation care are important contributors to outcome differences. The hospital that a patient is admitted to is not always intervenable—proximity frequently drives where emergency illnesses are treated—but other institutional factors represent targets for study and intervention. For instance, higher nurse-to-patient ratios are associated with improved adult IHCA outcomes.^8^ Centers offering extracorporeal membrane oxygenation may have improved chances of preventing or rescuing a patient from cardiac arrest. Ensuring access to high-quality IHCA care for all children is essential and could mitigate hospital-level differences.

Contrary to our hypothesis, public insurance was not associated with lower odds of survival in children receiving in-hospital CPR. This finding is similar to the pediatric sepsis report of Mitchell et al,^21^ in which they hypothesized that clinician lack of knowledge of patients’ insurance payers minimizes the impact of this “invisible variable.” However, other insurance payer (9.6% of cohort) was associated with higher rates of in-hospital mortality in children receiving CPR. This finding also mirrors those of Mitchell et al.^21^ While it is difficult to generalize based on this small, likely heterogeneous group, we agree with the authors of the cited sepsis study that while only a small percentage of US children are uninsured (5%),^22^ they represent a particularly vulnerable subgroup. Any insurance—private or public—may improve a child’s ability to receive adequate preventive and emergency health care and thereby result in lower severity of presenting illness, lower incidence of cardiac arrest, and lower rates of poor postarrest outcomes.

This study was motivated by the discrepancy between established racial disparities in mortality for many pediatric illnesses and a GWTG-R analysis that found differential rates of return of spontaneous circulation for Black children, but did not find a difference in hospital survival by race in pediatric IHCA.^9^ KID provided us with 10-fold more pediatric IHCAs without the inherent selection bias of a voluntary, resource-intense (ie, expensive) database such as GWTG-R. For example, in 2019, which is the most recent release year of KID data included in the study, there were 4949 unweighted CPR events (6545 weighted) at 4000 hospitals.^11^ In contrast, GWTG-R reported 598 pediatric IHCAs at 80 hospitals that year.^23^ In the current study, we were able to evaluate 27 332 CPR events with 6081 occurring among Black children compared with 2940 total IHCAs with 898 in Black children in the previous GWTG-R study.^9^ However, adult IHCA studies indicate that administrative datasets fail to include all cardiac arrests.^24^ Thus, KID, with its limited clinical information and reliance on procedural codes for case identification, presumably underestimates incidence but captures a higher absolute number of cases from a large, diverse group of hospitals. In contrast, GWTG-R captures substantially more clinical information and likely captures a higher proportion of a given hospital’s events but does so at a smaller cohort of sites that are voluntary members in a resuscitation quality improvement registry. The challenges of each database raise the importance of establishing a national IHCA registry.^25^

### Limitations

Our study should be interpreted in the context of several limitations. This is a cohort of children receiving CPR. As such, IHCA incidence was not investigated, and therefore differential prevention of IHCA based on our primary exposures would not be captured. Clinical data are limited and subject to misclassification in this administrative database. Identification of patients with IHCA relies on procedural code documentation, likely resulting in underreporting. Individual patients cannot be linked across hospitals using KID data; therefore, patients with IHCAs occurring at different hospitals during the study period could be included as distinct patients in the dataset. Race and ethnicity are a compound variable in KID with limited options and limited information on the mechanism of classifying individuals, likely resulting in improper classification and data loss (eg, determining Hispanic ethnicity separately from race or classifying individuals of multiple races). Insurance payer and median household income by zip code are imperfect proxies for economic status, with potentially superior markers of social determinants of health unavailable.^26^ We excluded 5208 patients who were missing a primary exposure or the outcome. While our race and ethnicity model was robust to multiple imputation for patients missing race and ethnicity (the largest group of missing data), there remains a possibility that this degree of missingness played a role in our findings. Since sites are assigned a unique hospital ID in each KID release year, some are likely represented several times in the analysis. Very few of the components of potential mechanisms for differences in outcomes, such as severity of illness, IHCA etiology, CPR quality metrics (eg, time to defibrillation), decisions to withdraw life-sustaining treatments, and institutional practices (eg, nursing ratios, resuscitation policies), are collected in the KID. Nevertheless, this large, population-based study with data from a diverse group of US hospitals provides a unique, important perspective on pediatric IHCA outcomes that is not available in other contemporary datasets on this scale.

## Conclusions

In this pediatric IHCA cohort study, we identified racial, ethnic, and insurance payer–based differences in outcomes for children in ethnic and racial minority groups. The mechanisms underlying differential pediatric IHCA outcomes must be elucidated. Our findings of lower odds of survival among children receiving CPR at hospitals with the highest proportions of Black or publicly insured patients may support the hypothesis that hospital resuscitation practices should be targeted for interventions to improve outcomes.

## Notes

Supplement 1. **eTable 1.** Relevant *International Classification of Disease* and Disease Related Group Codes **eTable 2.** Cohort Characteristics by Race or Ethnicity **eTable 3.** Cohort Characteristics by Insurance Status **eTable 4.** Sensitivity Analysis, Including Patients Transferred to Other Acute Care Hospitals **eTable 5.** Alternative Model: Adjusted Odds of In-Hospital Mortality After CPR, With Hospital Percentage of Admissions Occurring in Black Patients Included in the Model **eTable 6.** Adjusted Odds of In-Hospital Mortality After CPR by Treating Hospital’s Proportion of Publicly Insured Patients **eFigure 1.** Directed Acyclic Graph of Variables Included in the Model **eFigure 2**. Adjusted Odds of In-Hospital Mortality After CPR by Race or Ethnicity, Stratified by Geographic Region **eFigure 3**. Adjusted Odds of In-Hospital Mortality After CPR by Patient Race or Ethnicity, Stratified by Year **eAppendix 1.** Analyses of Patients Missing Primary Exposure(s) or Outcome **eAppendix 2.** Model Diagnostics for Primary Analyses

Supplement 2.
Data Sharing Statement
