# Supplementary material for: Race, Ethnicity, Insurance Payer, and Pediatric Cardiac Arrest Survival
Source: JAMA Netw Open. 2025 Sep 10;8(9):e2531213. doi: 10.1001/jamanetworkopen.2025.31213 (PMC12423836; doi:10.1001/jamanetworkopen.2025.31213)
Supplement: Supplement 1. — eTable 1. Relevant International Classification of Disease and Disease Related Group Codes eTable 2. Cohort Characteristics by Race or Ethnicity eTable 3. Cohort Characteristics by Insurance Status eTable 4. Sensitivity Analysis, Including Patients Transferred to Other Acute Care Hospitals eTable 5. Alternative Model: Adjusted Odds of In-Hospital Mortality After CPR, With Hospital Percentage of Admissions Occurring in Black Patients Included in the Model eTable 6. Adjusted Odds of In-Hospital Mortality After CPR by Treating Hospital’s Proportion of Publicly Insured Patients eFigure 1. Directed Acyclic Graph of Variables Included in the Model eFigure 2. Adjusted Odds of In-Hospital Mortality After CPR by Race or Ethnicity, Stratified by Geographic Region eFigure 3. Adjusted Odds of In-Hospital Mortality After CPR by Patient Race or Ethnicity, Stratified by Year eAppendix 1. Analyses of Patients Missing Primary Exposure(s) or Outcome eAppendix 2. Model Diagnostics for Primary Analyses [file jamanetwopen-e2531213-s001.pdf]

# Supplementary Online Content

O'Halloran AJ, Keim G, Gathers C-A, et al. Race, ethnicity, insurance payer, and survival of pediatric cardiac arrest. *JAMA Netw Open.* 2025;8(9):e2531213. doi:10.1001/jamanetworkopen.2025.31213

**eTable 1.** Relevant *International Classification of Disease* and Disease Related Group Codes
**eTable 2.** Cohort Characteristics by Race or Ethnicity
**eTable 3.** Cohort Characteristics by Insurance Status
**eTable 4.** Sensitivity Analysis, Including Patients Transferred to Other Acute Care Hospitals
**eTable 5.** Alternative Model: Adjusted Odds of In-Hospital Mortality After CPR, With Hospital Percentage of Admissions Occurring in Black Patients Included in the Model
**eTable 6.** Adjusted Odds of In-Hospital Mortality After CPR by Treating Hospital's Proportion of Publicly Insured Patients
**eFigure 1.** Directed Acyclic Graph of Variables Included in the Model
**eFigure 2**. Adjusted Odds of In-Hospital Mortality After CPR by Race or Ethnicity, Stratified by Geographic Region
**eFigure 3**. Adjusted Odds of In-Hospital Mortality After CPR by Patient Race or Ethnicity, Stratified by Year
**eAppendix 1.** Analyses of Patients Missing Primary Exposure(s) or Outcome
**eAppendix 2.** Model Diagnostics for Primary Analyses

This supplementary material has been provided by the authors to give readers additional information about their work.

© 2025 O'Halloran AJ et al. *JAMA Network Open*.

**eTable 1**. Relevant *International Classification of Disease* and Disease Related Group Codes

| |
|---|
| **Procedural Codes used to identify patients receiving CPR** |
| <u>ICD-9 (used for KID releases prior to 2016)</u> |
| 99.6 (cardiopulmonary resuscitation) |
| 99.63 (closed chest cardiac massage) |
| <u>ICD-10 (used for KID releases beginning in 2016)</u> |
| ESA005 (cardiac chest compression) |
| **Disease Related Group Codes used to identify obstetric illness category** |
| 540P.14 |
| 541P.14 |
| 542P.14 |
| 544P.14 |
| 545P.14 |
| 546P.14 |
| 560P.14 |
| 561M.14 |
| 563M.14 |
| 564M.14 |
| 565M.14 |
| 566M.14 |

© 2025 O’Halloran AJ et al. *JAMA Network Open*.

**eTable 2**. Cohort Characteristics by Race or Ethnicity

| | **Total** N=27,332 | **White** N=13,062 | **Black** N=6,081 | **Hispanic** N=5,123 | **Other** N=3,066 | **p-value** |
|---|---|---|---|---|---|---|
| **Age** | | | | | | <0.001 |
| ≤28 days | 6,366 (23.3%) | 2,819 (21.6%) | 1,574 (25.9%) | 1,115 (21.8%) | 858 (28.0%) | |
| 29 days to <1 year | 9,665 (35.4%) | 4,271 (32.7%) | 2,047 (33.7%) | 2,166 (42.3%) | 1,181 (38.5%) | |
| 1 year to <8 years | 4,867 (17.8%) | 2,618 (20.0%) | 942 (15.5%) | 853 (16.7%) | 454 (14.8%) | |
| ≥8 years | 6,434 (23.5%) | 3,354 (25.7%) | 1,518 (25.0%) | 989 (19.3%) | 573 (18.7%) | |
| **Female sex** | 11,976 (43.8%) | 5,623 (43.1%) | 2,730 (44.9%) | 2,252 (44.0%) | 1,371 (44.7%) | 0.066 |
| **Insurance Payer** | | | | | | <0.001 |
| Public | 13,689 (50.1%) | 4,988 (38.2%) | 4,107 (67.5%) | 3,182 (62.1%) | 1,412 (46.1%) | |
| Private | 11,010 (40.3%) | 7,045 (53.9%) | 1,453 (23.9%) | 1,242 (24.2%) | 1,270 (41.4%) | |
| Other | 2,633 (9.6%) | 1,029 (7.9%) | 521 (8.6%) | 699 (13.6%) | 384 (12.5%) | |
| **Complex chronic condition** | 5,277 (76.3%) | 2,181 (73.2%) | 1,389 (77.9%) | 995 (79.3%) | 712 (78.9%) | <0.001 |
| **Admission day is a weekend** | 5,528 (21.5%) | 2,424 (19.8%) | 1,356 (23.5%) | 1,097 (22.9%) | 651 (22.4%) | <0.001 |
| **Length of stay** (days, median [IQR]) | 4 (1-15) | 4 (1-13) | 4 (1-18) | 5 (1-18) | 5 (1-18) | <0.001 |
| **Patient Disposition** | | | | | | <0.001 |
| Discharged to Home or Self Care | 13,947 (51.0%) | 7,480 (57.3%) | 2,819 (46.4%) | 2,268 (44.3%) | 1,380 (45.0%) | |
| Other Transfer (SNIF or Intermediate Care) | 732 (2.7%) | 302 (2.3%) | 206 (3.4%) | 137 (2.7%) | 87 (2.8%) | |
| Discharged to Home Health Care | 1,534 (5.6%) | 730 (5.6%) | 356 (5.9%) | 288 (5.6%) | 160 (5.2%) | |
| Left Against Medical Advice | 16 (0.1%) | 7 (0.1%) | 3 (0.0%) | 3 (0.1%) | 3 (0.1%) | |
| In-Hospital Mortality | 11,080 (40.5%) | 4,531 (34.7%) | 2,694 (44.3%) | 2,421 (47.3%) | 1,434 (46.8%) | |
| Discharged alive, destination unknown | 23 (0.1%) | 12 (0.1%) | 3 (0.0%) | 6 (0.1%) | 2 (0.1%) | |
| **Total charges** (median [IQR]) | $36,747.00 (11,200.00-156,332.00) | $27,750.50 (9340.00-125,690.50) | $38,542.00 (11,539.00-157,147.00) | $58,437.00 (16,451.00-219,899.00) | 53,781.50 (14,208.50-209,961.00) | <0.001 |
| **Median household income quartile for patient zip code** | | | | | | <0.001 |
| 0-25th Percentile | 8,771 (33.0%) | 3,139 (24.6%) | 3,101 (52.0%) | 1,823 (37.1%) | 708 (24.4%) | |
| 26th-50th Percentile | 6,692 (25.2%) | 3,377 (26.4%) | 1,380 (23.2%) | 1,271 (25.8%) | 664 (22.9%) | |
| 51st-75th Percentile | 5,822 (21.9%) | 3,120 (24.4%) | 902 (15.1%) | 1,108 (22.5%) | 692 (23.8%) | |
| 76th-100th Percentile | 5,274 (19.9%) | 3,142 (24.6%) | 575 (9.7%) | 717 (14.6%) | 840 (28.9%) | |
| **Hospital Region** | | | | | | <0.001 |
| Northeast | 3,931 (14.4%) | 1,917 (14.7%) | 907 (14.9%) | 538 (10.5%) | 569 (18.6%) | |
| Midwest | 4,314 (15.8%) | 2,504 (19.2%) | 1,083 (17.8%) | 272 (5.3%) | 455 (14.8%) | |
| South | 12,310 (45.0%) | 6,071 (46.5%) | 3,467 (57.0%) | 1,852 (36.2%) | 920 (30.0%) | |
| West | 6,777 (24.8%) | 2,570 (19.7%) | 624 (10.3%) | 2,461 (48.0%) | 1,122 (36.6%) | |
| **Hospital Urban Location** | 23,285 (96.2%) | 10,348 (94.2%) | 5,327 (97.7%) | 4,869 (98.4%) | 2,741 (97.2%) | <0.001 |
| **Teaching Hospital** | 19,197 (79.3%) | 8,365 (76.1%) | 4,610 (84.6%) | 3,864 (78.1%) | 2,358 (83.6%) | <0.001 |

© 2025 O’Halloran AJ et al. *JAMA Network Open*.

| | | | | | | |
|---|---|---|---|---|---|---|
| **Hospital Size** | | | | | | <0.001 |
| Small | 3,651 (15.1%) | 1,900 (17.3%) | 710 (13.0%) | 702 (14.2%) | 339 (12.0%) | |
| Medium | 5,585 (23.1%) | 2,439 (22.2%) | 1,182 (21.7%) | 1,241 (25.1%) | 723 (25.6%) | |
| Large | 14,967 (61.8%) | 6,648 (60.5%) | 3,558 (65.3%) | 3,004 (60.7%) | 1,757 (62.3%) | |

© 2025 O'Halloran AJ et al. *JAMA Network Open*.

**eTable 3**. Cohort Characteristics by Insurance Status

| | **Total** N=27,332 | **Public** N=13,689 | **Private** N=11,010 | **Other** N=2,633 | **p-value** |
|---|---|---|---|---|---|
| **Age** | | | | | |
| ≤28 days | 6,366 (23.3%) | 3,477 (25.4%) | 2,309 (21.0%) | 580 (22.0%) | <0.001 |
| 29days to < 1 year | 9,665 (35.4%) | 4,993 (36.5%) | 3,825 (34.7%) | 847 (32.2%) | |
| 1 year to <8 years | 4,867 (17.8%) | 2,496 (18.2%) | 1,891 (17.2%) | 480 (18.2%) | |
| ≥ 8 years | 6,434 (23.5%) | 2,723 (19.9%) | 2,985 (27.1%) | 726 (27.6%) | |
| **Female sex** | 11,976 (43.8%) | 6,092 (44.5%) | 4,756 (43.2%) | 1,128 (42.9%) | 0.074 |
| **Race** | | | | | <0.001 |
| White | 13,062 (47.8%) | 4,988 (36.4%) | 7,045 (64.0%) | 1,029 (39.1%) | |
| Black | 6,081 (22.2%) | 4,107 (30.0%) | 1,453 (13.2%) | 521 (19.8%) | |
| Hispanic | 5,123 (18.7%) | 3,182 (23.2%) | 1,242 (11.3%) | 699 (26.5%) | |
| Other | 3,066 (11.2%) | 1,412 (10.3%) | 1,270 (11.5%) | 384 (14.6%) | |
| **Complex chronic condition** | 5,277 (76.3%) | 3,008 (77.4%) | 1,786 (74.4%) | 483 (76.2%) | 0.024 |
| **Admission day is a weekend** | 5,528 (21.5%) | 2,859 (21.9%) | 2,090 (20.5%) | 579 (23.3%) | 0.003 |
| **Length of stay** (days, median [IQR]) | 4 (1-15) | 5 (1-18) | 4 (1-13) | 3 (1-10) | <0.001 |
| **Patient Disposition** | | | | | |
| Discharged to Home or Self Care | 13,947 (51.0%) | 6,719 (49.1%) | 6,063 (55.1%) | 1,165 (44.2%) | <0.001 |
| Other Transfer (SNIF or Intermediate Care) | 732 (2.7%) | 426 (3.1%) | 247 (2.2%) | 59 (2.2%) | |
| Discharged to Home Health Care | 1,534 (5.6%) | 852 (6.2%) | 584 (5.3%) | 98 (3.7%) | |
| Left Against Medical Advice | 16 (0.1%) | 10 (0.1%) | 6 (0.1%) | 0 (0.0%) | |
| In-Hospital Mortality | 11,080 (40.5%) | 5,669 (41.4%) | 4,104 (37.3) | 1,307 (49.6%) | |
| Discharged alive, destination unknown | 23 (0.1%) | 13 (0.1%) | 6 (0.1%) | 4 (0.2%) | |
| **Total charges** (median [IQR]) | 36,747.00 (11200-156332) | 42,494.00 (12028-178884) | 32,286.00 (10577-140080) | 29,751.00 (10671-121858) | <0.001 |
| **Median household income quartile for patient zip code** | | | | | <0.001 |
| 0-25th Percentile | 8,771 (33.0%) | 5,786 (43.2%) | 2,183 (20.3%) | 802 (33.1%) | |
| 26th-50th Percentile | 6,692 (25.2%) | 3,642 (27.2%) | 2,372 (22.1%) | 678 (28.0%) | |
| 51st-75th Percentile | 5,822 (21.9%) | 2,525 (18.9%) | 2,743 (25.5%) | 554 (22.9%) | |
| 76th-100th Percentile | 5,274 (19.9%) | 1,435 (10.7%) | 3,452 (32.1%) | 387 (16.0%) | |
| **Hospital Region** | | | | | |
| Northeast | 3,931 (14.4%) | 1,681 (12.3%) | 1,957 (17.8%) | 293 (11.1%) | <0.001 |
| Midwest | 4,314 (15.8%) | 2,209 (16.1%) | 1,759 (16.0%) | 346 (13.1%) | |
| South | 12,310 (45.0%) | 6,664 (48.7%) | 4,494 (40.8%) | 1,152 (43.8%) | |
| West | 6,777 (24.8%) | 3,135 (22.9%) | 2,800 (25.4%) | 842 (32.0%) | |
| **Hospital Urban Location** | 23,285 (96.2%) | 11,727 (95.8%) | 9,336 (96.8%) | 2,222 (96.0%) | <0.001 |
| **Teaching Hospital** | 19,197 (79.3%) | 9,825 (80.2%) | 7,605 (78.9%) | 1,767 (76.3%) | 0.010 |
| **Hospital Size** | | | | | |
| Small | 3,651 (15.1%) | 1,755 (14.3%) | 1,547 (16.0%) | 349 (15.1%) | <0.001 |
| Medium | 5,585 (23.1%) | 2,756 (22.5%) | 2,260 (23.4%) | 569 (24.6%) | |
| Large | 14,967 (61.8%) | 7,736 (63.2%) | 5,834 (60.5%) | 1,397 (60.3%) | |

© 2025 O'Halloran AJ et al. *JAMA Network Open*.

**eTable 4.** Sensitivity Analysis, Including Patients Transferred to Other Acute Care Hospitals

| **Race or Ethnicity** | **aOR (95% CI)** | **P-value** |
|---|---|---|
| White (reference) | 1 | |
| Black | 1.20 (1.08 – 1.33) | 0.001 |
| Hispanic | 1.18 (1.06 – 1.31) | 0.003 |
| Other | 1.33 (1.17 – 1.50) | <0.001 |

aOR: adjusted odds ratio
Other race or ethnicity includes Asian or Pacific Islander, Native American, and Other categorizations in KID. Hospital (size, region, teaching, rural/urban distinction, KID cohort year) and patient-level factors (age, presence of chronic renal insufficiency or malignancy, income quartile by zip code, insurance payer, biologic sex) were considered fixed effects and treating hospital was considered a random effect.

**eTable 5**. Alternative Model: Adjusted Odds of In-Hospital Mortality After CPR, With Hospital Percentage of Admissions Occurring in Black Patients Included in the Model

| **Race or Ethnicity** | **aOR (95% CI)** | **P-value** |
|---|---|---|
| White (reference) | 1 | |
| Black | 1.14 (1.02 – 1.27) | 0.026 |
| Hispanic | 1.15 (1.03 – 1.29) | 0.013 |
| Other | 1.41 (1.23 – 1.62) | <0.001 |

OR: odds ratio; aOR: adjusted odds ratio
Other race includes Asian or Pacific Islander, Native American, and Other categorizations in KID. Hospital (size, region, teaching, rural/urban distinction, KID cohort year, and percentage of admissions occurring in Black patients) and patient-level factors (age, presence of chronic renal insufficiency or malignancy, income quartile by zip code, insurance payer, biologic sex) were considered fixed effects and treating hospital was considered a random effect.

**eTable 6.** Adjusted Odds of In-Hospital Mortality After CPR by Treating Hospital's Proportion of Publicly Insured Patients

| **Proportion of Publicly Insured Patients at Treating Hospital** (n=number of hospitals) | **aOR (95% CI)** | **P-value** |
|---|---|---|
| 0-40.9% (n=1068) | 1 (Base) | |
| 40.91-53.5% (n=1007) | 0.93 (0.79 - 1.09) | 0.374 |
| 53.51-64.1% (n=937) | 1.18 (1.00 - 1.39) | 0.047 |
| >64.11% (n=1159) | 1.21 (1.02 - 1.42) | 0.028 |

CPR: cardiopulmonary resuscitation; aOR: adjusted odds ratio
Includes data from 2003-2019. Hospital (size, region, teaching, rural/urban distinction, KID cohort year) and patient-level factors (age, presence of chronic renal insufficiency or malignancy, income quartile by zip code, race or ethnicity, biologic sex) level factors were considered fixed effects and treating hospital was considered a random effect.

© 2025 O'Halloran AJ et al. *JAMA Network Open*.

**eFigure 1.** Directed Acyclic Graph of Variables Included in the Model

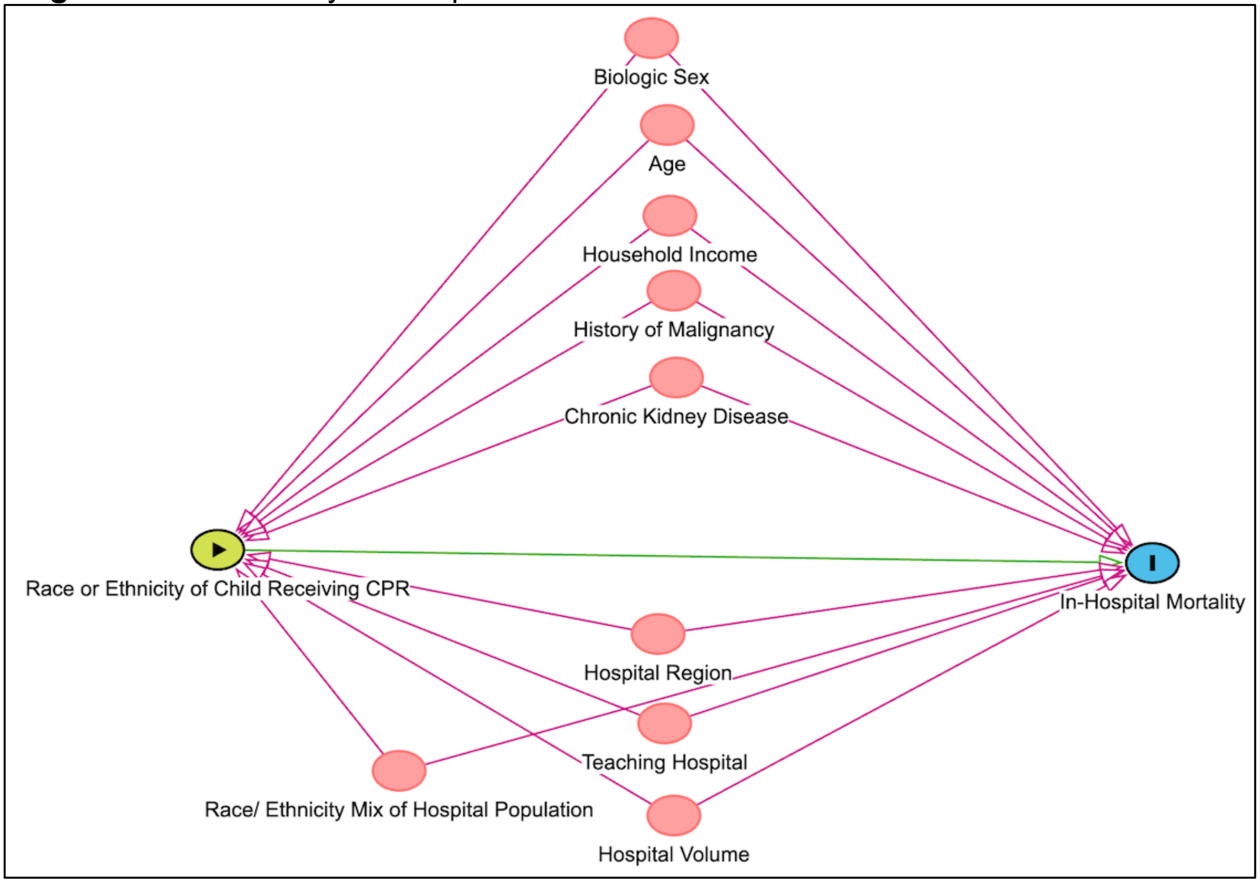


© 2025 O’Halloran AJ et al. *JAMA Network Open*.

**eFigure 2.** Adjusted Odds of In-Hospital Mortality After CPR by Race or Ethnicity, Stratified by Geographic Region

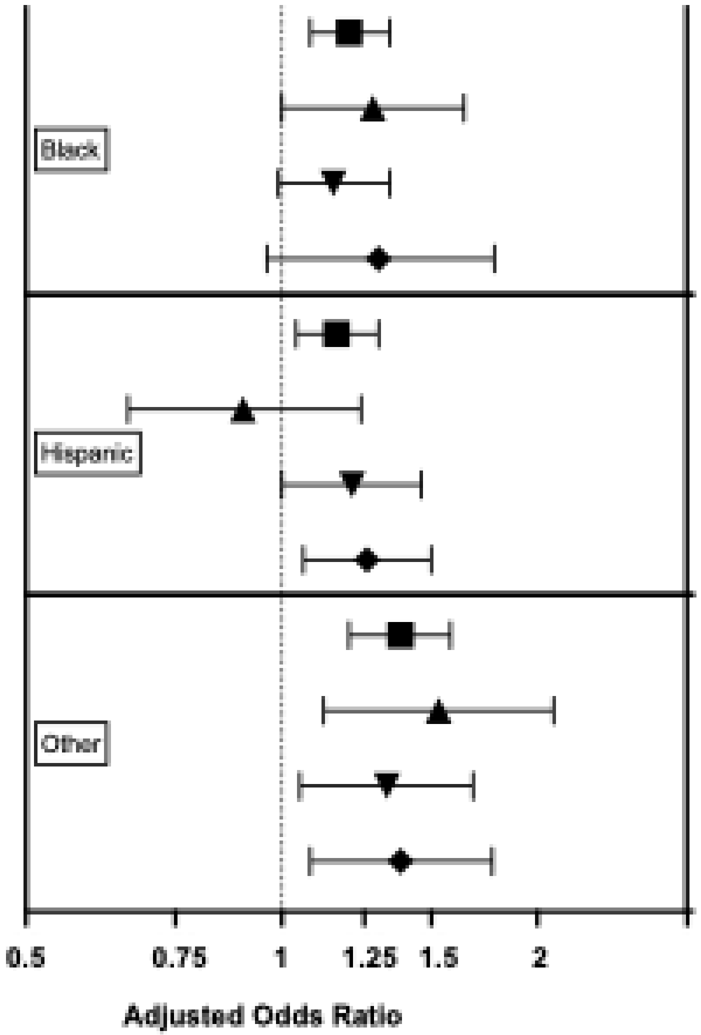


| Region | aOR (95%CI) |
|---|---|
| Northeast | 1.20 (1.08, 1.34) |
| Midwest | 1.27 (1.0, 1.64) |
| South | 1.15 (0.99, 1.34) |
| West | 1.30 (0.96, 1.78) |
| Northeast | 1.16 (1.04, 1.30) |
| Midwest | 0.90 (0.66, 1.24) |
| South | 1.21 (1.0, 1.46) |
| West | 1.26 (1.06, 1.5) |
| Northeast | 1.38 (1.20, 1.58) |
| Midwest | 1.53 (1.12, 2.09) |
| South | 1.33 (1.05, 1.68) |
| West | 1.38 (1.08, 1.77) |

Forest plot of adjusted odds ratio for in-hospital mortality after cardiopulmonary resuscitation by patient race or ethnicity, stratified by geographic region.
aOR: adjusted odds ratio
Other race includes Asian or Pacific Islander, Native American, and Other categorizations in KID. Hospital (size, region, teaching, rural/urban distinction, KID cohort year) and patient-level factors (age, presence of chronic renal insufficiency or malignancy, income quartile by zip code, insurance payer, biologic sex) were considered fixed effects and treating hospital was considered a random effect.

© 2025 O’Halloran AJ et al. *JAMA Network Open*.

**eFigure 3**. Adjusted Odds of In-Hospital Mortality After CPR by Patient Race or Ethnicity, Stratified by Year

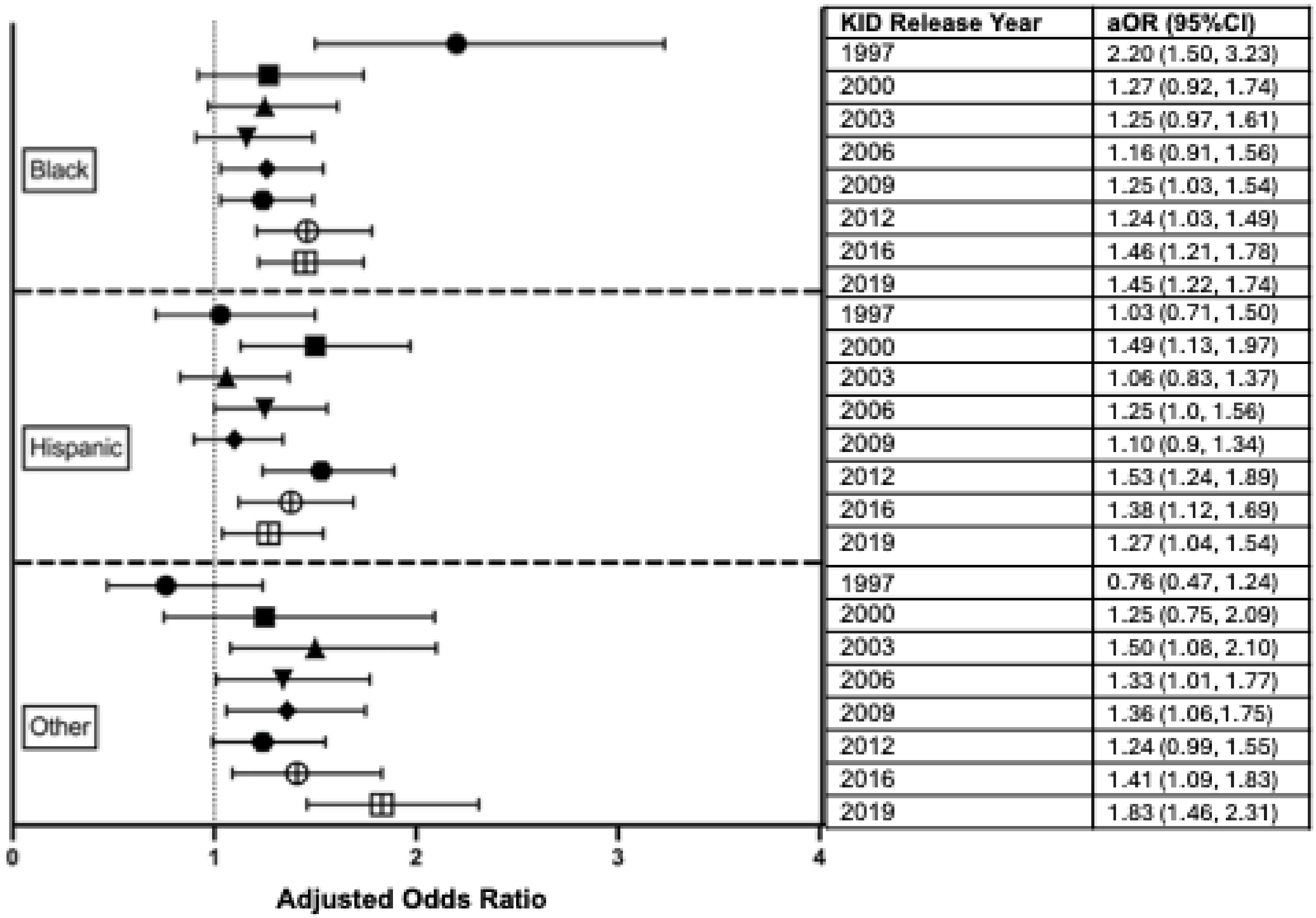


| KID Release Year | aOR (95%CI) |
|---|---|
| 1997 | 2.20 (1.50, 3.23) |
| 2000 | 1.27 (0.92, 1.74) |
| 2003 | 1.25 (0.97, 1.61) |
| 2006 | 1.16 (0.91, 1.56) |
| 2009 | 1.25 (1.03, 1.54) |
| 2012 | 1.24 (1.03, 1.49) |
| 2016 | 1.46 (1.21, 1.78) |
| 2019 | 1.45 (1.22, 1.74) |
| 1997 | 1.03 (0.71, 1.50) |
| 2000 | 1.49 (1.13, 1.97) |
| 2003 | 1.06 (0.83, 1.37) |
| 2006 | 1.25 (1.0, 1.56) |
| 2009 | 1.10 (0.9, 1.34) |
| 2012 | 1.53 (1.24, 1.89) |
| 2016 | 1.38 (1.12, 1.69) |
| 2019 | 1.27 (1.04, 1.54) |
| 1997 | 0.76 (0.47, 1.24) |
| 2000 | 1.25 (0.75, 2.09) |
| 2003 | 1.50 (1.08, 2.10) |
| 2006 | 1.33 (1.01, 1.77) |
| 2009 | 1.36 (1.06,1.75) |
| 2012 | 1.24 (0.99, 1.55) |
| 2016 | 1.41 (1.09, 1.83) |
| 2019 | 1.83 (1.46, 2.31) |

Forest plot of adjusted odds ratio for in-hospital mortality after cardiopulmonary resuscitation by patient race or ethnicity, stratified by KID release year.
aOR: adjusted odds ratio
Other race includes Asian or Pacific Islander, Native American, and Other categorizations in KID. Hospital (size, region, teaching, rural/urban distinction, KID cohort year) and patient-level factors (age, presence of chronic renal insufficiency or malignancy, income quartile by zip code, insurance payer, biologic sex) were considered fixed effects and treating hospital was considered a random effect.

© 2025 O'Halloran AJ et al. *JAMA Network Open*.

**eAppendix 1.** Analyses of Patients Missing Primary Exposure(s) or Outcome

## Patient and Hospital Characteristics of Eligible and Excluded Patients

| | **Patients with CPR Code** | **Eligible Patients** | **Key Exposure or Outcome Variable Missing** |
|---|---|---|---|
| | n = 36,779 | n = 31,571 | n = 5,208 |
| **Age** | | | |
| ≤28 days | 9,295 (25.3%) | 8,221 (26.0%) | 1,074 (20.6%) |
| 29 days to <1 year | 14,141 (38.4%) | 11,593 (36.7%) | 2548 (48.9%) |
| 1 year to <8 years | 5,714 (15.5%) | 5,083 (16.1%) | 631 (12.1%) |
| ≥8 years | 7,629 (20.7%) | 6,674 (21.2%) | 955 (18.3%) |
| **Sex** | | | |
| Female | 16,168 (44.0%) | 13,809 (43.8%) | 2,359 (45.4%) |
| **Race or Ethnicity** | | | |
| Black | 6,911 (18.8%) | 6,879 (21.8%) | 32 (0.6%) |
| Hispanic | 5,931 (16.1%) | 5,924 (18.8%) | 7 (0.1%) |
| White | 15,351 (41.7%) | 15,252 (48.3%) | 99 (1.9%) |
| Other | 3,522 (9.6%) | 3,516 (11.1%) | 6 (0.1%) |
| **Insurance Payer** | | | |
| Public | 18,167 (49.4%) | 15,889 (50.3%) | 2,278 (43.7%) |
| Private | 14,902 (40.5%) | 12,671 (40.1%) | 2,231 (42.8%) |
| Other | 3,557 (9.7%) | 3,011 (9.5%) | 546 (10.5%) |
| **Complex chronic condition** | 6,887 (73.5%) | 6,147 (73.7%) | 740 (72.0%) |
| **Admission day is a weekend** | () | () | () |
| **Length of stay** (days; median [IQR]) | 3 (1-13) | 3 (1-14) | 1 (1-10) |
| **Patient Disposition** | | | |
| Discharged to Home or Self Care | 16,051 (43.6%) | 13,947 (44.2%) | 2,104 (40.4%) |
| Other Transfer (SNF or Intermediate Care) | 859 (2.3%) | 732 (2.3%) | 127 (2.4%) |
| Discharged to Home Health Care | 1,787 (4.9%) | 1,534 (4.9%) | 253 (4.9%) |
| Left Against Medical Advice | 21 (0.1%) | 16 (0.1%) | 5 (0.1%) |
| In-Hospital Mortality | 12,956 (35.2%) | 11,080 (35.1%) | 1,876 (36.0%) |
| Discharged alive, destination unknown | 70 (0.2%) | 23 (0.1%) | 47 (0.9%) |
| **Median household income quartile for patient zip code** | | | |
| 0-25th percentile | 11,351 (30.9%) | 10,040 (31.8%) | 1,311 (25.2%) |
| 26th-50th percentile | 9,280 (25.2%) | 7,833 (24.8%) | 1,447 (27.8%) |
| 51st-75th percentile | 8,023 (21.8%) | 6,807 (21.6%) | 1,216 (23.3%) |
| 76th-100th percentile | 7,128 (19.4%) | 6,035 (19.1%) | 1,093 (21.0%) |
| **Hospital Region** | | | |
| Northeast | 5,050 (13.7%) | 4,623 (14.6%) | 427 (8.2%) |
| Midwest | 7,463 (20.3%) | 5,241 (16.6%) | 2,222 (42.7%) |
| South | 15,033 (40.9%) | 13,624 (43.2%) | 1,409 (27.1%) |

© 2025 O’Halloran AJ et al. *JAMA Network Open*.

| | | | |
|---|---|---|---|
| West | 9,233 (25.1%) | 8,083 (25.6%) | 1,150 (22.1%) |
| **Teaching Hospital** | 24,641 (73.8%) | 21,157 (74.5%) | 3,484 (70.1%) |
| **Hospital Size** | | | |
| Small | 5,123 (13.9%) | 4,359 (13.8%) | 764 (14.7%) |
| Medium | 7,862 (21.4%) | 6,741 (21.4%) | 1,121 (21.5%) |
| Large | 20,399 (55.5%) | 17,315 (54.8%)) | 3,084 (59.2%) |

IQR: interquartile range; SNF: skilled nursing facility

Other race includes Asian or Pacific Islander, Native American, and Other categorizations in KID.

Hospital size in the database takes into account the hospital’s region, urban-rural designation

© 2025 O’Halloran AJ et al. *JAMA Network Open*.

**Multiple Imputation for Missing Race or Ethnicity Data**

We performed multiple imputation by chained equations to test the robustness of our complete case analysis to missing race/ethnicity variables. A multinomial-logit model was specified for the race/ethnicity exposure including the outcome (mortality), all covariates in the analytic model, and a reduced set of hospital indicators to preserve site-level differences: hospitals with fewer than 10 non-missing exposure observations were collapsed into a single "other-small" category. Each completed data set was analyzed with the original mixed-effects logistic regression—random intercept for the original hospital identifier and robust, hospital-clustered standard errors—and the results were combined with Rubin's rules.

Across the imputations, within-imputation variance far exceeded between-imputation variance for every coefficient (average relative increase in variance = 0.02), the largest fraction of missing information was 0.25 ("Other" race), and all relative efficiencies exceeded 0.95, indicating that imputations were sufficient. After adjustment, children identified as Black (OR = 1.18, 95 % CI 1.06–1.31; $p = 0.003$), Hispanic (OR = 1.15, 95 % CI 1.03–1.29; $p = 0.015$) or Other (OR = 1.34, 95 % CI 1.15–1.56; $p < 0.001$) had higher odds of in-hospital death than White children, virtually unchanged from the complete-case analysis. The model F-test remained significant ($F = 14.7$, $p < 0.001$) and all other covariate associations and hospital-level effects mirrored those observed previously, confirming that the race-mortality association is robust to the treatment of missing race/ethnicity data.

© 2025 O'Halloran AJ et al. *JAMA Network Open*.

**eAppendix 2.** Model Diagnostics for Primary Analyses

Race or Ethnicity Model

Initially, we tested the additive value of hospital level effects, finding that addition of this random-intercept using a mixed-effect model substantially improved performance reducing both the AIC (29,156 vs. 30,923) and BIC (29,386 vs. 31,145). Next, we quantified hospital-level heterogeneity using the intraclass correlation coefficient, finding 26 % of the unexplained variance in the odds of death remained attributable to differences between hospitals (ICC = 0.26, 95 % CI 0.22–0.30), highlighting substantial clustering at the hospital level. The model showed good discrimination (AUC = 0.81) and reduced mean squared error compared with a non-informative benchmark (Brier score = 0.176 vs 0.236). Although average predictions were 2 percentage-points too high (Spiegelhalter z = −22.2), calibration within risk strata was acceptable (Reliability-small = 0.005).

Insurance Payer Model

In the insurance-based model, substantial between-hospital heterogeneity persisted, with 26 % of the residual variance in mortality attributable to hospitals (ICC = 0.26, 95 % CI 0.22–0.30). The mixed-effects specification greatly out-performed the fixed-effects alternative (AIC = 29,156 vs 30,923; BIC = 29,387 vs 31,146), confirming the need for a hospital random intercept. Predictive performance remained strong: the model achieved good discrimination (AUC = 0.81) and a Brier score of 0.176, representing a 25 % reduction in mean-squared error relative to a non-informative benchmark. Average calibration was slightly optimistic (mean predicted risk 39.6 % vs observed 37.7 %; Spiegelhalter z = –22.2), but calibration within risk strata was excellent (reliability-small = 0.005), and resolution metrics (Sanders = 0.171; Murphy = 0.064) showed the model separated high- and low-risk patients effectively. Together, these diagnostics indicate that, even with primary insurer as the exposure, the model fits well, discriminates accurately, and retains clinically acceptable calibration while accounting for meaningful hospital-level clustering.

© 2025 O’Halloran AJ et al. *JAMA Network Open*.
