# Supplementary material for: Race, Ethnicity, Insurance Payer, and Pediatric Cardiac Arrest Survival
Source: JAMA Netw Open. 2025 Sep 10;8(9):e2531213. doi: 10.1001/jamanetworkopen.2025.31213 (PMC12423836; doi:10.1001/jamanetworkopen.2025.31213)
Supplement: Supplement 2. — Data Sharing Statement [file jamanetwopen-e2531213-s002.pdf]

Data Sharing Statement

O'Halloran. Race, Ethnicity, Insurance Payer, and Pediatric Cardiac Arrest Survival. *JAMA Netw Open*. Published September 10, 2025. doi:10.1001/jamanetworkopen.2025.31213

Data
Data available: No

Additional Information
Explanation for why data not available: The data that support the findings of this study are available from the Healthcare Cost and Utilization Project's (HCUP) Kids' Inpatient Database. Restrictions apply to the availability of these data, which were used under the appropriate Data Use Agreement for this study. Data are available from https://hcup-us.ahrq.gov/kidoverview.jsp with the permission of HCUP.
